# Supplementary material for: MetaRibo-Seq measures translation in microbiomes
Source: Nat Commun. 2020 Jun 29;11:3268. doi: 10.1038/s41467-020-17081-z (PMC7324362; doi:10.1038/s41467-020-17081-z)
Supplement: Supplementary file 10 — Supplementary Data 7 [file 41467_2020_17081_MOESM10_ESM.zip › File2/Confidence_High_Taxonomy/305453_out.krona.html]

Javascript must be enabled to view this page.

members
magnitude
magnitudeUnassigned
count
unassigned
taxon
rank

305453\_out

36

2
36
superkingdom

phylum
1239
36

class
186801
36

36
186802
order

186803
36
family

genus
36
841
1

SRS893252\_contig\_number\_contig-100\_13581.57693


SRS011084\_contig\_number\_31742SRS013158\_contig\_number\_contig-100\_673.40690SRS014459\_contig\_number\_5894SRS014923\_contig\_number\_33415SRS015190\_contig\_number\_15187SRS015960\_contig\_number\_15195SRS016335\_contig\_number\_contig-100\_1822.197683SRS017821\_contig\_number\_4550SRS019267\_contig\_number\_4987SRS022137\_contig\_number\_1318SRS022609\_contig\_number\_6658SRS024549\_contig\_number\_contig-100\_29.30224SRS043001\_contig\_number\_10192SRS043411\_contig\_number\_6569SRS044535\_contig\_number\_contig-100\_19649.64039SRS045713\_contig\_number\_contig-100\_471.471SRS046068\_contig\_number\_1581SRS050925\_contig\_number\_33852SRS063040\_contig\_number\_contig-100\_259.256332SRS063370\_contig\_number\_contig-100\_25443.25444SRS065504\_contig\_number\_9452SRS074964\_contig\_number\_contig-100\_236.236SRS076929\_contig\_number\_17730SRS077641\_contig\_number\_20773SRS097889\_contig\_number\_6841SRS101433\_contig\_number\_8370SRS105153\_contig\_number\_23259SRS142781\_contig\_number\_9442SRS143070\_contig\_number\_12846SRS143780\_contig\_number\_15788SRS148091\_contig\_number\_4496SRS148159\_contig\_number\_contig-100\_333.331107SRS148721\_contig\_number\_10804SRS149244\_contig\_number\_4756SRS893378\_contig\_number\_21210
166486
35
species
